# Detection of Glioblastoma Subclinical Recurrence Using Serial Diffusion Tensor Imaging

**DOI:** 10.3390/cancers12030568

**Published:** 2020-02-29

**Authors:** Yan Jin, James W. Randall, Hesham Elhalawani, Karine A. Al Feghali, Andrew M. Elliott, Brian M. Anderson, Lara Lacerda, Benjamin L. Tran, Abdallah S. Mohamed, Kristy K. Brock, Clifton D. Fuller, Caroline Chung

**Affiliations:** 1 Department of Radiation Oncology, the University of Texas MD Anderson Cancer Center, Houston, TX 77030, USA; yjinz@ucla.edu (Y.J.); jamesran92@gmail.com (J.W.R.); elhalawani.hesham@gmail.com (H.E.); kal1@mdanderson.org (K.A.A.F.); andrew.elliott@mdanderson.org (A.M.E.); lcalvarez@mdanderson.org (L.L.); bltran@mdanderson.org (B.L.T.); asmohamed@mdanderson.org (A.S.M.); cdfuller@mdanderson.org (C.D.F.); 2 The University of Texas Medical Branch, Galveston, TX 77555, USA; 3 Department of Imaging Physics, the University of Texas MD Anderson Cancer Center, Houston, TX 77030, USA; bmanderson@mdanderson.org (B.M.A.); kkbrock@mdanderson.org (K.K.B.)

**Keywords:** glioblastoma, radiation therapy, recurrence, MRI, diffusion tensor imaging

## Abstract

Glioblastoma is an aggressive brain tumor with a propensity for intracranial recurrence. We hypothesized that tumors can be visualized with diffusion tensor imaging (DTI) before they are detected on anatomical magnetic resonance (MR) images. We retrospectively analyzed serial MR images from 30 patients, including the DTI and T1-weighted images at recurrence, at 2 months and 4 months before recurrence, and at 1 month after radiation therapy. The diffusion maps and T1 images were deformably registered longitudinally. The recurrent tumor was manually segmented on the T1-weighted image and then applied to the diffusion maps at each time point to collect mean FA, diffusivities, and neurite density index (NDI) values, respectively. Group analysis of variance showed significant changes in FA (*p* = 0.01) and NDI (*p* = 0.0015) over time. Pairwise t tests also revealed that FA and NDI at 2 months before recurrence were 11.2% and 6.4% lower than those at 1 month after radiation therapy (*p* < 0.05), respectively. Changes in FA and NDI were observed 2 months before recurrence, suggesting that progressive microstructural changes and neurite density loss may be detectable before tumor detection in anatomical MR images. FA and NDI may serve as non-contrast MR-based biomarkers for detecting subclinical tumors.

## 1. Introduction

Glioblastoma (GBM) is the most common primary malignant brain tumor among adults [1]. Despite treatment with surgical resection, radiation therapy (RT), chemotherapy, and temozolomide, many patients experience intracranial tumor recurrence, and prognosis remains poor, with a median survival time of only 12−15 months [2,3,4]. In current clinical practice, the onset of recurrence is defined in terms of changes in traditional images, such as contrast-enhanced computed tomography or structural T1-weighted post-contrast magnetic resonance imaging (T1 MRI), followed if possible by histologic verification. However, given the high frequency of recurrence in GBM and the poor survival rate thereafter, early detection of recurrence is key to facilitating timely medical treatment and prolonging survival.

Glioma cells have been shown to migrate into healthy brain tissues along white matter (WM) tracts [5,6]. The molecular mechanism underlying this phenomenon is complex and contains multiple steps, including tumor cell adhesion to components of the extracellular matrix and modification to their compositions, migration by breaking adherence attachments to the matrix, and the degradation of matrix proteins by secreting enzymes [7]. This suggests that examining the integrity of WM tracts within the potential recurrent regions before visible, enhancing tumor recurrence may help further elucidate the mechanism of recurrence.

Diffusion-weighted imaging is a noninvasive MRI technique in which measurements of water diffusion in tissues [8] provide biological and clinically relevant information about the integrity of WM that cannot be provided by other imaging modalities. Diffusion tensor imaging (DTI) applies a mathematical elliptical model to represent the diffusion characteristics at each voxel [9]. A variety of diffusion-derived feature maps, such as fractional anisotropy (FA), mean diffusivity (MD), axial diffusivity (AD), and radial diffusivity (RD), can be extracted from DTI images and have provided complementary information about the integrity of WM in several neurologic diseases, such as multiple sclerosis [10], Parkinson disease [11], and Alzheimer disease [12].

In brain tumors, DTI has previously been used to assess WM disruptions to identify tumor grades [13,14], distinguish tumor tissue from peritumoral tissue [15], and study the patterns of shape (isotropic versus anisotropic) in recurrence [16]. Reduced FA has been linked with decreased fiber density index near tumors in patients with GBM [17]. However, details of how WM tracts are disrupted during the clinical course of recurrence are yet to be elucidated. We hypothesized that tumor cells in the recurrent regions cause microstructural disruptions in WM integrity and these disruptions can be visualized by DTI before tumors can be detected on conventional anatomical MRI. To test this hypothesis, we conducted a retrospective longitudinal study to evaluate whether changes in a series of DTI feature maps precede the clinical onset of recurrence. We also used an advanced diffusion analysis technique, neurite orientation dispersion and density imaging (NODDI) [18], to provide further details on how microstructures in WM are affected at the microscale level.

## 2. Materials and Methods

### 2.1. Patients and Image Acquisition

Under an institutional review board-approved protocol (PA18-1113), we extracted MRI data from 30 patients treated for GBM at the University of Texas MD Anderson Cancer Center who had experienced recurrence after RT. All patients had anatomical T1 and DTI brain imaging at four time points: at 1 month after RT (baseline), 4 months before recurrence, 2 months before recurrence, and at recurrence. The average time between baseline and recurrence was 13 months with a standard deviation of 12 months.

All scans were obtained according to a standard protocol developed for GBM follow-up at the University of Texas MD Anderson Cancer Center and were acquired on 1.5 T/3T MRI scanners (GE Medical Systems) with axial orientation. T1 images had a 256 × 256/512 × 512 in-plane acquisition matrix and a voxel resolution of 0.86 × 0.86 × 6.5 mm^3^ (TR/TE = 550/8 ms)/0.43 × 0.43 × 5 mm^3^ (TR/TE = 967/12 ms). DTI images consisted of 28 volumes with a 256 × 256 in-plane acquisition matrix and a voxel resolution of 0.86 × 0.86 × 6.5 mm^3^ (TR/TE = 1000/99 ms)/0.86 × 0.86 × 5 mm^3^ (TR/TE = 10,250/94 ms), including one non-diffusion sensitization volume, i.e., T2-weighted b0 volume, and 27 diffusion-weighted volumes (b = 1200 s/mm^2^).

### 2.2. Image Preprocessing

T1 images were first skull-stripped with the open source skull stripping toolkit LABEL [19]. DTI images were also skull-stripped with Brain Extraction Tool in the Functional Magnetic Resonance Imaging of the Brain (FMRIB) Software Library (FSL; https://fsl.fmrib.ox.ac.uk/fsl/fslwiki) [20,21] and then corrected for eddy current-induced distortions and subject movements using EDDY [22] in FSL.

### 2.3. Microstructural Features with NODDI

We used NODDI, an advanced diffusion analysis technique, to provide additional details about the microstructural features the images represent. Dendrites and axons, known as neural processes or neurites, are the basic units for brain circuits. Using NODDI to investigate variations in branching complexity (i.e., density and orientation) allowed us to characterize changes in microstructural integrity in terms of recurrence. Specifically, NODDI provides three indices to describe such changes: neurite density index (NDI), orientation dispersion index (ODI), and free water fraction (FWF) [23]. NDI and ODI quantify the density and angular variation of neurites, and FWF describes the contamination of tissues by free water at the microstructural level [23].

### 2.4. Longitudinal Image Processing Pipeline

Our longitudinal image processing pipeline consisted of the following six steps: Step 1. Diffusion Feature Extraction: The following diffusion-derived feature maps were extracted from the DTI images of each patient at each time point by using DTIFIT in FSL: fractional anisotropy (FA), mean diffusivity (MD), axial diffusivity (AD), and radial diffusivity (RD). Next, we calculated the microstructural indices (NDI, ODI, and FWF) at each voxel of the DTI images with the NODDI Matlab Toolbox (http://mig.cs.ucl.ac.uk/index.php?n=Tutorial.NODDImatlab). Step 2. Longitudinal Image Alignment: We applied advanced normalization tools (http://stnava.github.io/ANTs/) [24] to register each patient’s T1 images at 1 month after RT, 4 months before recurrence, and 2 months before recurrence to the T1 image obtained at recurrence. Because these T1 images had been acquired at different times, a nonlinear (warping) registration algorithm was used to ensure the best alignment effect. Step 3. Multi-Modality Image Alignment: At each time point, each patient’s DTI images were co-registered using linear (rigid) registration with the corresponding T1 images, as both images were acquired during the same imaging session. Step 4. GTV Manual Segmentation: An experienced radiation oncologist manually segmented the tumor recurrence volume on the T1 images at the time of recurrence. Step 5. GTV Transfer: For each patient, the recurrent tumor volume was applied to the T1 images at the previous three time points with the deformation fields generated by nonlinear registrations in step 2. The recurrent tumor volume was then transferred to the corresponding DTI images with the affine matrix generated by linear registrations in step 3. The quality of registration and GTV transfer at steps 2–5 was manually inspected. Step 6. Mean Feature Calculation: The mean values of all diffusion features (FA, MD, AD, and RD) and microstructural features (NDI, ODI, and FFW) within the transferred and original recurrent GTV regions at the four time points were calculated.

### 2.5. Statistical Analysis

We compared all mean DTI features at the four time points using analysis of variance (ANOVA) to examine potential changes between these time points. Findings are reported as F ratios, i.e., the ratio of the between-group variance to the within-group variance. If any changes were detected, *t* tests were applied to further investigate changes occurring at each pair of time points.

## 3. Results

### 3.1. Diffusion Feature Maps

**Figure 1:**
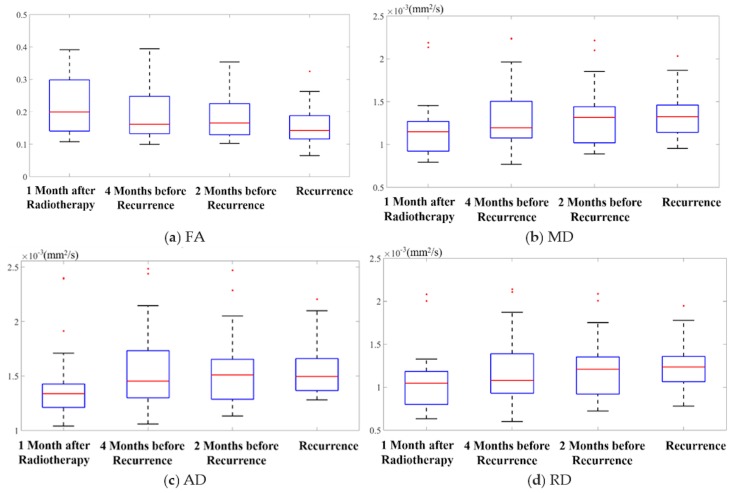
Box plots of the mean values of (**a**) fractional anisotropy (FA), (**b**) mean diffusivity (MD), (**c**) axial diffusivity (AD), and (**d**) radial diffusivity (RD) within recurrent tumor regions over the four time points. The five bars in the box plots indicate, from bottom to top, the minimum, first quartile, median, third quartile, and the maximum. The red dots indicate outliers.

Mean FA values inside the regions of recurrence over the four time points are shown in box plots in Figure 1a. The ANOVA revealed statistically significant differences between time points (F = 3.4, *p* = 0.01), with a downward trend over time. To further examine which groups were different from one another, we used pairwise t tests to compare the four time points. After Bonferroni correction, the mean FA value at recurrence was significantly lower than those at the three prior time points (*p* < 0.05), with a decrease of 30.8% (95% confidence interval-CI: (18.2%, 43.0%)), 22.9% (95% CI: (11.0%, 38.9%)), and 19.2% (95% CI: (8.7%, 35.4%)) from the farthest to the nearest time point, respectively. Moreover, the mean FA value at 2 months before recurrence was also 11.2% lower than that at 1 month after RT, i.e., the baseline (*p* < 0.05). At the individual level, 14 and 18 out of 30 patients showed a lower mean FA value at 4 months before recurrence and 2 months before recurrence, compared to baseline, respectively.

Findings from the ANOVA of the mean values of the other feature maps (MD, AD, and RD) over time are shown in Figure 1b–d. No statistically significant differences were detected in these feature maps between time points: for MD, F = 1.38, *p* = 0.25; for AD, F = 0.99, *p* = 0.40; and for RD, F = 1.57, *p* = 0.20. Although these values were not statistically different, they demonstrated a trend of increasing values over time, as is evident in those figures. Further pairwise t tests showed that the mean values of MD, AD, and RD at 2 months before recurrence were 9.5% (95% CI: (4.0%, 15.4%)), 7.8% (95% CI: (2.5%, 13.0%)), and 10.9% (95% CI: (4.8%, 17.1%)) higher than those at baseline, respectively (*p* < 0.05 after Bonferroni correction). At the individual level, 18, 16, and 18 patients had a higher mean MD, AD, and RD value at 2 months before recurrence, compared to baseline, respectively.

Changes in these features (FA, MD, AD, and RD) may indicate that WM degeneration occurred at recurrent tumor regions before the tumor became visible on T1.

### 3.2. Microstructural Features

**Figure 2:**
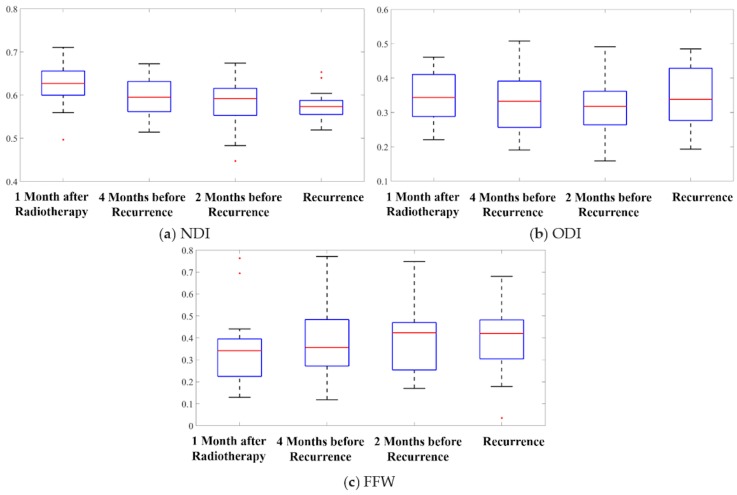
Box plots of the mean values of (**a**) neurite density index (NDI), (**b**) orientation dispersion index (ODI), and (**c**) free water fraction [FWF] within recurrent tumor regions over the four time points. The five bars in the box plots indicate, from bottom to top, the minimum, first quartile, median, third quartile, and the maximum. The red dots indicate outliers.

To investigate which WM microstructural components were affected by tumor recurrence, we used the same statistical tests of the mean values of NDI, ODI, and FFW (obtained with the NODDI toolbox) at the regions of recurrence over time. The NDI results were similar to those of the FA findings (Figure 2a). ANOVA revealed differences among the four time points (F = 5.47, *p* = 0.0015), with a downward trend over time. Pairwise t tests showed that the mean NDI value at recurrence was significantly lower than baseline and 4 months before recurrence, a relative change of 11.9% (95% CI: (4.3%, 19.5%)), and 7.2% (95% CI: (0.4%, 15.6%)), respectively (*p* < 0.05 after Bonferroni correction). Furthermore, compared to baseline, the mean NDI values at 4 months before recurrence were 4.2% (95% CI: (1.5%, 6.9%)) lower and the mean NDI values at 2 months before recurrence were 6.4% (95% CI: (3.4%, 9.5%)) lower (*p* < 0.05 after Bonferroni correction). At the individual level, 21 and 20 patients demonstrated a reduced mean NDI value at 4 months before recurrence and 2 months before recurrence, compared to baseline, respectively.

The ANOVA showed no differences in ODI over time (F = 1.16, *p* = 0.33) (Figure 2b). Similarly, FWF did not change over time according to the ANOVA (F = 0.93, *p* = 0.43) (Figure 2c).

The observation of a change in NDI but not in ODI before recurrence suggests that microstructural changes in WM due to tumor recurrence may have resulted from a reduction in neurite density rather than a disruption in orientation distribution.

### 3.3. Qualitative Comparison

**Figure 3:**
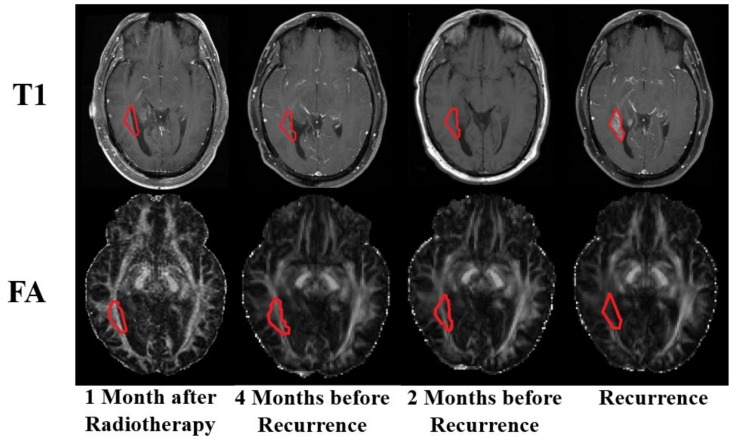
Serial T1 and fractional anisotropy (FA) images of a representative patient. Red outlines indicate the location of the recurrent tumor.

T1 MRI and FA images of a representative patient at each of the four time points are shown in Figure 3. The recurrent tumor (circled in red) was not visible on the three conventional T1 images before the evident time point of recurrence, but WM deterioration at the recurrent region was visibly appreciable on the FA images at 4 months and 2 months before recurrence. Notably, this recurrence appeared at the intersection of the internal capsule and geniculocalcarine tract (optic radiation), which are major WM tracts in the brain. These images corroborate our hypothesis that tumor cells may cause WM disruptions that may be evident on DTI images before the actual tumor is visible in T1 images.

### 3.4. Radiation Therapy versus Recurrence

It is known that RT would cause dose-dependent white matter damage around treatment areas [25,26]. Therefore, the values of diffusion parameters such as FA would be affected after treatment. In order to distinguish the longitudinal reduction of FA in the study was caused by recurrence or RT, we selected 9 patients from our dataset whose recurrent intervals were longer than 12 months. And we calculated the mean FA values of the recurrence regions at an additional time point—6 months after RT. We performed pairwise t tests at between 6 months after RT and 1 month after RT, 4 months before recurrence, 2 months before recurrence, and recurrence, respectively.

**Figure 4:**
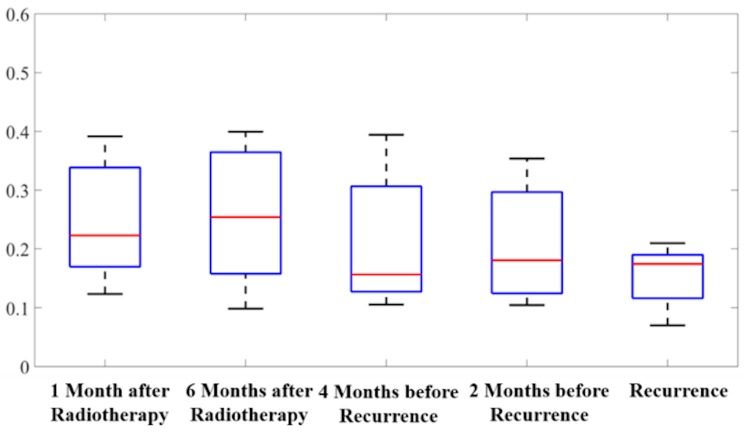
Box plots of the mean values of fractional anisotropy (FA) within recurrent tumor regions over the five time points for selected 9 patients. The five bars in the box plots indicate, from bottom to top, the minimum, first quartile, median, third quartile, and the maximum.

**Figure 5:**
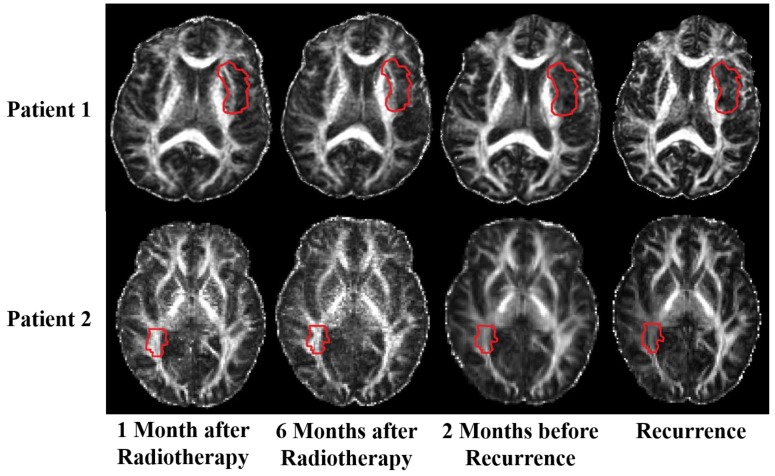
Serial fractional anisotropy (FA) images of two representative patients at four time points, including 6 months after radiation therapy. Red outlines indicate the location of the recurrent tumor.

There was no statistically significant change in the mean FA values between 1 month after RT and 6 months after RT (*p* = 0.80, 95% CI: (−12.7%, 12.7%)). However, compared with those at 6 months after RT, the mean FA values were significantly lower at 2 months before recurrence (*p* = 0.04, 95% CI: (0.8%, 28.3%)) and at recurrence (*p* = 0.008, 95% CI: (12.7%, 63.4%)). The mean FA values inside the regions of recurrence over the five time points are shown in box plots in Figure 4. Qualitatively, we showed the FA images at 1 month after RT, 6 months after RT, 2 months before recurrence, and recurrence from two representative patients in Figure 5. The red contours outlined the recurrent tumor regions. White matter inside the contours seemed to be intact at 1 month and 6 months after RT, whereas degeneration could be observed at 2 months before recurrence and at recurrence. Both quantitative and qualitative results indicate that the change in FA in our study may result from tumor recurrence instead of radiation-induced damage.

## 4. Discussion

Although T1 weighted post contrast MRI remains the gold standard for predicting GBM recurrence, other imaging modalities such as positron emission tomography (PET) and perfusion MRI have been utilized to assist and improve the diagnostic accuracy of tumor recurrence [27,28]. In particular, DTI has the unique capability of displaying information on tissue cellularity, microstructures, and microvasculature by means of noninvasive images [29,30,31] and thus is increasingly investigated for cancer diagnosis [32] and the prediction of treatment response [33]. Numerous feature maps can be generated from DTI to measure changes in different diffusion properties. For example, FA represents the directionality or the degree of diffusion anisotropy, whereas MD reflects the overall diffusion properties. AD describes the diffusion properties along a particular direction (the principal axis), whereas RD accounts for the diffusion in the other two directions perpendicular to the principal direction [12].

Although DTI has been studied in several types of cancer [34], there are limited studies in GBM. For example, Chang et al. [35] compared the MD values in post-surgical images with those of post-recurrence images in tumor and surrounding tissues to predict tumor recurrence. The current study is one of the first to investigate the utility of DTI for detecting possible changes in WM microstructure before the appearance of a recurrent tumor on conventional MRI.

Our results support our hypothesis that the disruption of WM integrity near the site of recurrence in patients with GBM can precede the appearance of an enhancing tumor on structural MRI. When WM degenerates, the diffusion directionality decreases. When fiber tracts disassociate, water molecules can move freely without obstruction in any direction, and diffusivity usually increases. In general, FA and MD usually move in opposite directions. In our study, we expected that FA values would be lower at recurrence than that at baseline, and that MD would increase as a result of WM degeneration caused by underlying tumor growth. Our findings reflect these expectations, and we found similar trends of increasing values for AD and RD over time. Chang et al. [35] found that MD values were lower inside the tumor than in the surrounding peritumoral region, whereas we found that MD values tended to increase within the tumor region from baseline to recurrence. The pattern of rising MD values, as seen in our study, is consistent with findings in other neurologic diseases, such as Alzheimer disease, where MD values increased in the regions of WM degeneration [12]. Other studies of diffusion MRI brain tumors have speculated that MD is an important marker for tumor response assessment as it is negatively associated with cell density or cellularity, which is a critical measure for tumor progression [34,36,37]. However, our results suggest that the directionality feature (FA) was more sensitive to changes in WM that predicted for GBM recurrence than the other diffusivity features (MD, AD, and RD) suggesting that FA may also be a useful non-contrast imaging biomarker of GBM recurrence.

To further explore changes at the microstructural level and possibly provide more insight into tumor invasion along WM tracts, we used NODDI, a recently developed imaging technique that provides information on specific voxel-wise microstructural substrates via three components, each with values ranging from 0 to 1: NDI describes relative neurite density, ODI quantifies relative angular or morphologic variations, and FWF defines the aggregated free water portion. In the current study, NDI could distinguish recurrence at different stages of development, but the other two components could not. Because changes in FA can result from a combination of NDI and ODI [16], we suspect that the decrease in FA in our study may have been caused by a decrease in NDI rather than in ODI. This in turn suggests that neurite loss (as opposed to neurite morphologic change) may be the main pathologic process for tumor invasion in GBM recurrence, a finding that warrants histologic confirmation in future studies. Not surprisingly, similar patterns of change have also been observed in other neurodegenerative diseases, such as frontotemporal lobar degeneration and amyotrophic lateral sclerosis [23].

In previous studies, diffusion parameters demonstrated significant changes from their baseline values in the areas affected by RT at 6 months after RT [25,26]. In our study, the mean FA values of the region of interest did not show obvious changes between 6 months after RT and baseline (i.e. 1 month after RT) whereas they did show changes between 6 months after RT and 2 months before recurrence in the region of tumor recurrence. Although the small sample size may limit the statistical power, it does imply that the changes we observed from our dataset may be due to white matter deterioration caused by early tumor recurrence rather than radiation.

Despite its promising findings, the current study had several limitations. Our sample size was limited by the difficulty of retrospectively identifying patients treated with concurrent RT and temozolomide with complete sets of serial imaging data at the proposed study time points. With regards to the imaging consistency, the serial DTI images for patients included in this retrospective review were acquired at varying magnet strengths, both 1.5T and 3T without any consistent pattern over time. Although the pattern of image acquisition at varying magnet strengths was random, it may have impacted our results. Additionally, although all the registration results were manually checked after the automated, deformable image registration algorithm, any residual errors in the registration of the GTV to the diffusion images across all four time points have the potential to affect the quantitative measures within these GTV regions before recurrence.

As this is a small retrospective study, the intent of the study was to investigate whether there were suggestive findings that DTI changes may help detect earlier pathological changes prior to traditional anatomical imaging in areas of GBM recurrence based on the hypothesis that an active tumor in a region may lead to the disruption of white matter integrity. For the purpose of this early signal finding study, group analysis enabled statistical analysis. At the individual patient level, we provide several examples of patients with demonstrated changes in DTI at a time point sooner than the clinical onset of recurrence. In our patient cohort, 18 patients of the 30 total group demonstrated changes in FA 2 months prior to confirmed tumor recurrence. Based on these findings, we conclude that DTI changes are promising early biomarkers to help localize areas of GBM recurrence, but further larger prospective studies would need to be done to validate our findings before any clinical implementation could be considered.

## 5. Conclusions

The results from this retrospective longitudinal study suggest that the disruption of WM integrity near the site of recurrence in patients with GBM can precede the appearance of enhancing tumor on structural MRI. The FA values on images obtained before recurrence were different from those in the baseline images, and the microstructural feature NDI decreased significantly between 2–4 months before recurrence and baseline, suggesting that underlying progressive microstructural changes may precede the appearance of enhancing tumor. We further found that FA was more sensitive than other diffusion-derived features such as MD, AD, and RD for detecting subclinical tumor presence. FA may therefore be useful as an imaging biomarker to predict GBM recurrence, and DTI can provide important complementary information to conventional structural MRI to assist in the clinical diagnosis of recurrence. Our results also suggest that the underlying mechanism for FA reduction may be neurite loss rather than changes in neurite morphology. Future prospective studies of larger cohorts of patients with GBM using a more uniform imaging protocol and more detailed analysis are planned to evaluate the role of DTI in detecting subclinical GBM in a normal-appearing brain and, potentially, to refine the definition of clinical target volume for RT planning and for predicting GBM recurrence.
